# Supplementary material for: Spatiotemporal changes in along-tract profilometry of cerebellar peduncles in cerebellar mutism syndrome
Source: Neuroimage Clin. 2022 Mar 30;35:103000. doi: 10.1016/j.nicl.2022.103000 (PMC9421471; doi:10.1016/j.nicl.2022.103000)
Supplement: Supplementary data 1 [file mmc1.docx]

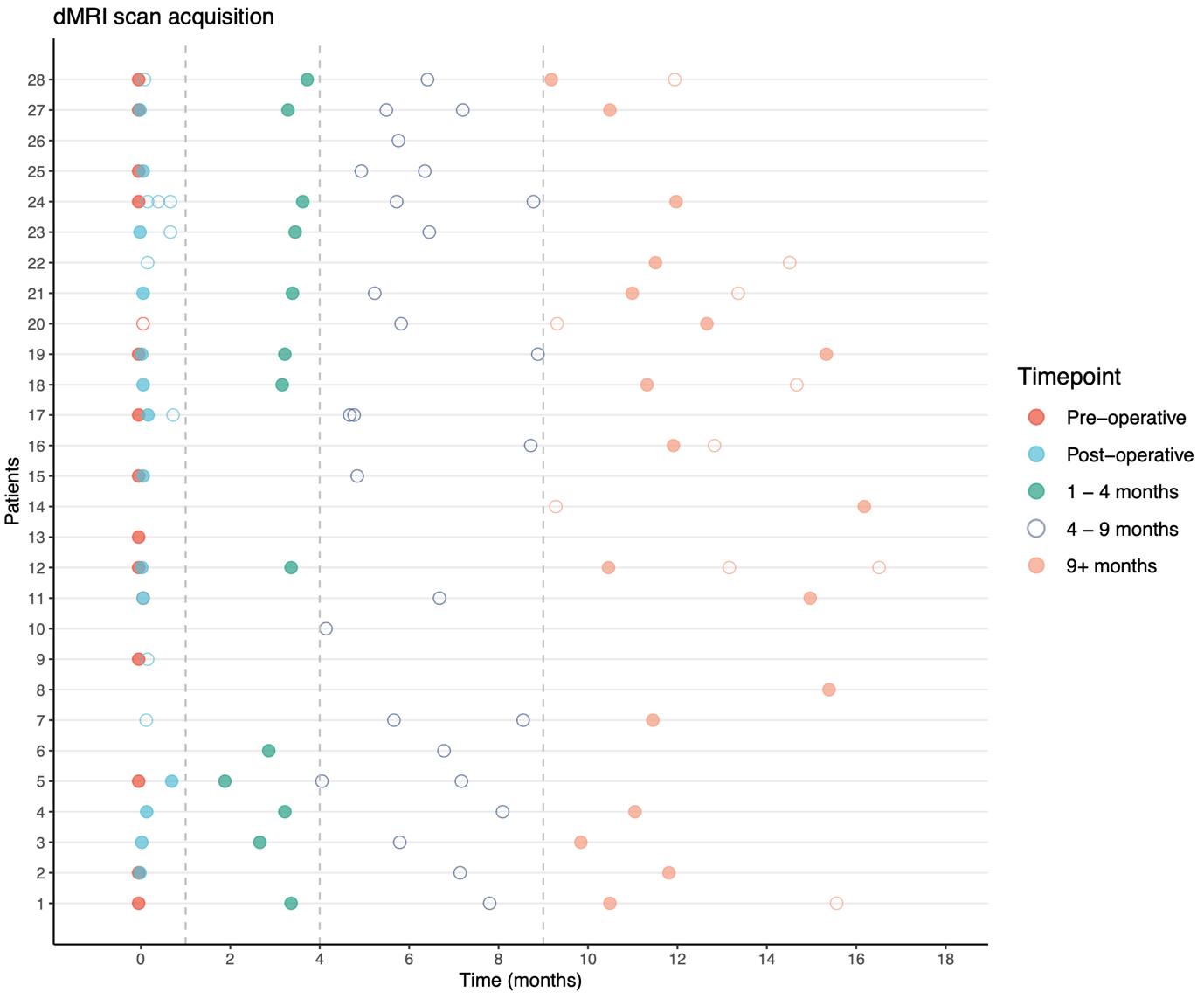


Supplementary Figure 1. Diffusion MRI scan acquisition for eligible subjects (n=28). Scans were grouped into four timepoints: pre-operative, post-operative, early follow-up (1-4 months) and late follow-up (>9 months). Scans acquired between 4 and 9 months post-operatively (dark blue circles) were not included in the analysis. Filled circles indicate scan inclusion in analysis.

**
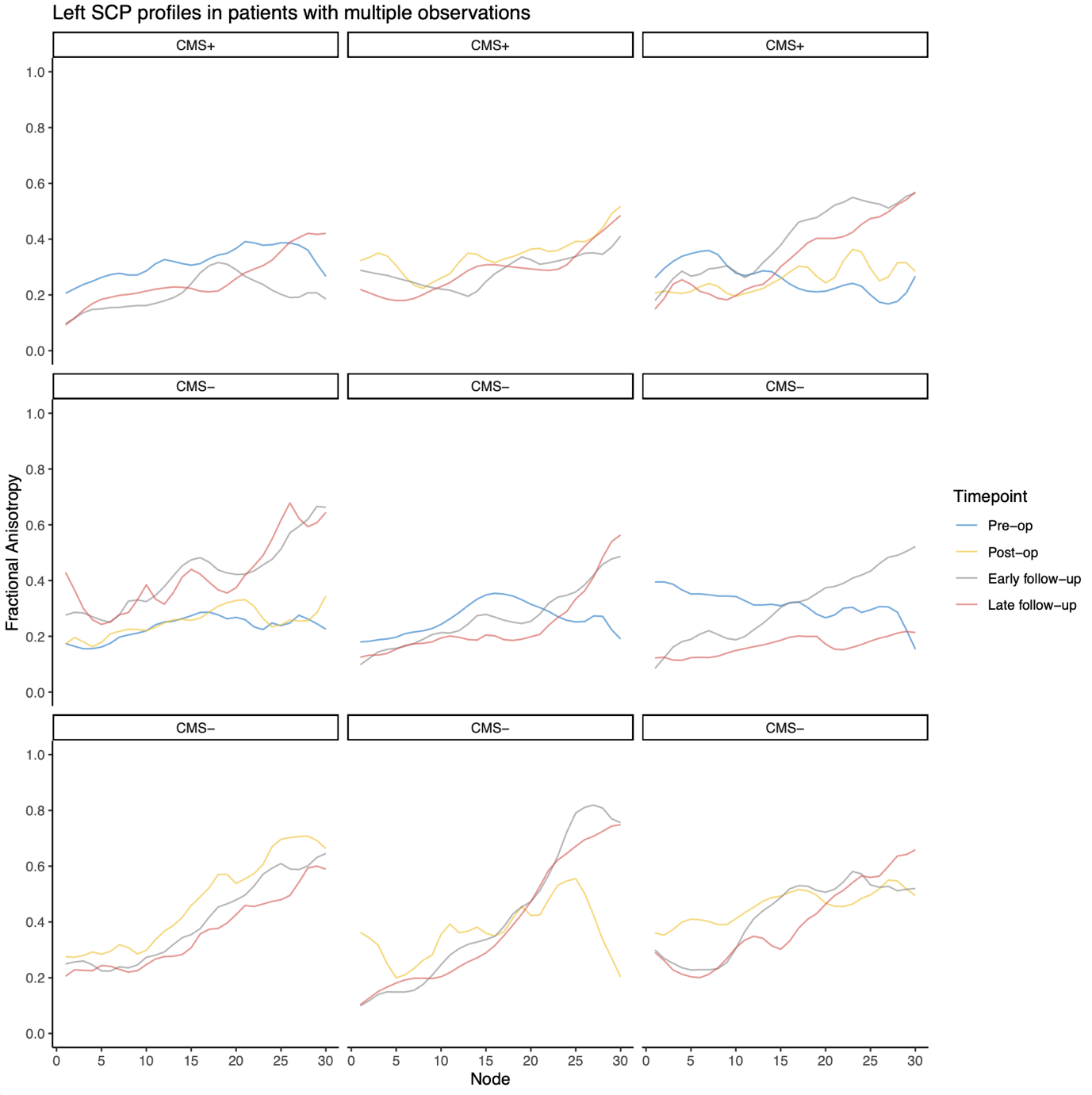
**

Supplementary Figure 2. Along-tract profilometry of the left SCP in patients with viable tracts at more than three timepoints.

Supplementary Table 1. Values indicate multiple-comparison corrected *p* values. Cells in bold indicate p<0.05.

|  | Tract segment | Pre-op | Post-op | Early FU | Late FU |
| --- | --- | --- | --- | --- | --- |
| Left SCP | | | | | |
| CMS |  | 0.957 | 0.341 | 0.209 | 0.162 |
| Tract segment |  | **0.005** | **0.000382** | 0.163 | **0.028** |
|  | 1st vs 2nd | 0.946 | 0.389 | 0.292 | 0.715 |
|  | 1st vs 3rd | **0.0137** | **0.0262** | 0.292 | 0.524 |
|  | 2nd vs 3rd | **0.0137** | 0.0979 | 0.978 | 0.524 |
| CMS:segment |  | 0.798 | 0.102 | **0.006** | **0.006** |
|  | 1st | 0.763 | 0.829 | 0.477 | 0.909 |
|  | 2nd | 0.702 | 0.166 | 0.145 | 0.201 |
|  | 3rd | 0.858 | 0.1 | **0.0421** | **0.0381** |
| Right SCP | | | | | |
| CMS |  | 0.479 | 0.435 | 0.848 | 0.648 |
| Tract segment |  | **0.045** | 0.185 | 0.05 | 0.126 |
|  | 1st vs 2nd | 0.842 | 0.627 | 0.538 | 0.936 |
|  | 1st vs 3rd | 0.242 | 0.627 | 0.829 | 0.404 |
|  | 2nd vs 3rd | 0.242 | 0.627 | 0.538 | 0.404 |
| CMS:segment |  | 0.977 | 0.225 | 0.338 | 0.52 |
|  | 1st | 0.718 | 0.893 | 0.795 | 0.999 |
|  | 2nd | 0.358 | 0.405 | 0.578 | 0.816 |
|  | 3rd | 0.221 | 0.173 | 0.625 | 0.297 |
| Left ICP | | | | | |
| CMS |  | 0.985 | 0.793 | 0.344 | 0.617 |
| Tract segment |  | 0.1 | **0.00335** | **0.039** | **0.033** |
|  | 1st vs 2nd | 0.469 | **0.00308** | 0.451 | 0.142 |
|  | 1st vs 3rd | 0.646 | **0.00177** | 0.103 | 0.142 |
|  | 2nd vs 3rd | 0.489 | 0.657 | 0.239 | 0.949 |
| CMS:segment |  | 0.641 | 0.449 | 0.607 | 0.511 |
|  | 1st | 0.737 | 0.856 | 0.348 | 0.828 |
|  | 2nd | 0.918 | 0.636 | 0.9 | 0.618 |
|  | 3rd | 0.729 | 0.57 | 0.233 | 0.37 |
| Right ICP | | | | | |
| CMS |  | 0.314 | 0.31 | 0.332 | 0.202 |
| Tract segment |  | 0.059 | **0.003** | **0.006** | 0.054 |
|  | 1st vs 2nd | 0.53 | 0.203 | 0.339 | 0.664 |
|  | 1st vs 3rd | 0.57 | 0.182 | 0.128 | 0.395 |
|  | 2nd vs 3rd | 0.429 | 0.686 | 0.391 | 0.371 |
| CMS:segment |  | 0.254 | 0.934 | **0.019** | 0.771 |
|  | 1st | 0.0532 | 0.461 | 0.0512 | 0.309 |
|  | 2nd | 0.497 | 0.329 | 0.379 | 0.218 |
|  | 3rd | 0.771 | 0.266 | 0.523 | 0.3 |
| MCP |  |  |  |  |  |
| CMS |  | 0.298 | 0.355 | 0.488 | 0.117 |
| Tract segment |  | 0.259 | 0.516 | 0.394 | 0.753 |
|  | 1st vs 2nd | 0.226 | 0.847 | 0.595 | 0.999 |
|  | 1st vs 3rd | 0.698 | 0.847 | 0.589 | 0.999 |
|  | 2nd vs 3rd | 0.222 | 0.847 | 0.595 | 0.999 |
| CMS:segment |  | 0.539 | 0.47 | 0.781 | 0.432 |
|  | 1st | 0.312 | 0.738 | 0.427 | 0.186 |
|  | 2nd | 0.834 | 0.575 | 0.503 | 0.22 |
|  | 3^rd^ | 0.397 | 0.114 | 0.985 | 0.134 |

Values indicate multiple-comparison corrected *p* values. Cells in bold indicate p<0.05
